# Fitts’ Law in the Control of Isometric Grip Force With Naturalistic Targets

**DOI:** 10.3389/fpsyg.2018.00560

**Published:** 2018-04-26

**Authors:** Zachary C. Thumser, Andrew B. Slifkin, Dylan T. Beckler, Paul D. Marasco

**Affiliations:** ^1^Laboratory for Bionic Integration, Department of Biomedical Engineering, Lerner Research Institute, Cleveland Clinic, Cleveland, OH, United States; ^2^Research Service, Louis Stokes Cleveland VA Medical Center, Cleveland, OH, United States; ^3^Department of Psychology, Cleveland State University, Cleveland, OH, United States; ^4^Advanced Platform Technology Center of Excellence, Louis Stokes Cleveland VA Medical Center, Cleveland, OH, United States

**Keywords:** Fitts’ law, force, grip, grasp, isometric, vision, tactile, feedback

## Abstract

Fitts’ law models the relationship between amplitude, precision, and speed of rapid movements. It is widely used to quantify performance in pointing tasks, study human-computer interaction, and generally to understand perceptual-motor information processes, including research to model performance in isometric force production tasks. Applying Fitts’ law to an isometric grip force task would allow for quantifying grasp performance in rehabilitative medicine and may aid research on prosthetic control and design. We examined whether Fitts’ law would hold when participants attempted to accurately produce their intended force output while grasping a manipulandum when presented with images of various everyday objects (we termed this the implicit task). Although our main interest was the implicit task, to benchmark it and establish validity, we examined performance against a more standard visual feedback condition via a digital force-feedback meter on a video monitor (explicit task). Next, we progressed from visual force feedback with force meter targets to the same targets without visual force feedback (operating largely on feedforward control with tactile feedback). This provided an opportunity to see if Fitts’ law would hold without vision, and allowed us to progress toward the more naturalistic implicit task (which does not include visual feedback). Finally, we changed the nature of the targets from requiring explicit force values presented as arrows on a force-feedback meter (explicit targets) to the more naturalistic and intuitive target forces implied by images of objects (implicit targets). With visual force feedback the relation between task difficulty and the time to produce the target grip force was predicted by Fitts’ law (average *r*^2^ = 0.82). Without vision, average grip force scaled accurately although force variability was insensitive to the target presented. In contrast, images of everyday objects generated more reliable grip forces without the visualized force meter. In sum, population means were well-described by Fitts’ law for explicit targets with vision (*r*^2^ = 0.96) and implicit targets (*r*^2^ = 0.89), but not as well-described for explicit targets without vision (*r*^2^ = 0.54). Implicit targets should provide a realistic see-object-squeeze-object test using Fitts’ law to quantify the relative speed-accuracy relationship of any given grasper.

## Introduction

Fitts’ law provides a model relating task difficulty to the time required to execute an action. In particular, Fitts’ law describes the strong positive linear relation between the index of difficulty (ID) and movement time. In essence, the ID (measured in binary units or bits of information) is the logarithm of the ratio of twice the movement distance or amplitude requirement divided by the target width, where target width specifies the tolerance for movement endpoint variability. Performance is described by the slope of the linear regression or by the mean ID-to-movement-time ratio (throughput), both of which provide an index of system information processing speed or capacity that can be measured in bits/s, although the latter may be preferable when comparing two or more experimental conditions (Soukoreff and MacKenzie, 2004).

The vast majority of studies on Fitts’ law utilize isotonic contractions of upper extremity muscles that drive displacement of an end effector (e.g., a hand) over different distances to targets of different widths, where the target boundaries specify the amount of tolerable endpoint variability. In other words, most studies on Fitts’ law require participants to point to a target. Despite the general focus on the control of visually guided manual aiming (e.g., Plamondon and Alimi, 1997), Fitts’ law has been shown to apply to a wide range of task conditions (Beilock et al., 2004; Duarte and Freitas, 2005; Ivanoff et al., 2008; Yamaguchi et al., 2013).

In the current study, we examined how Fitts’ law applied to the production of grasp forces under different visual feedback and target display conditions. Grasping an object is analogous to pointing to a target in a number of ways; indeed, Smeets and Brenner (1999) describe grasping as “nothing more than pointing with the thumb and finger toward selected positions on the surface of the object,” but parallels extend beyond the reach-and-grasp phase to the isometric force production phase of establishing and maintaining grip force upon the grasped object. For example, just as pointing to a farther object may require the generation of more muscular force to move the hand over a longer distance, the force required to grip and lift an object increases as its weight increases. Thus, there is a desired target force in grasping actions, just as in an isotonic pointing task there is a desired target position that requires force regulation. In addition, different objects permit different degrees of variability in the production of those forces. For example, some objects may be more durable, and therefore can withstand larger variations in grip forces, but fragile objects may have lower tolerance for grip force variations. Thus, there is a tolerable range of force variability, just as in a pointing task there are boundaries to the target area.

**FIGURE 1:**
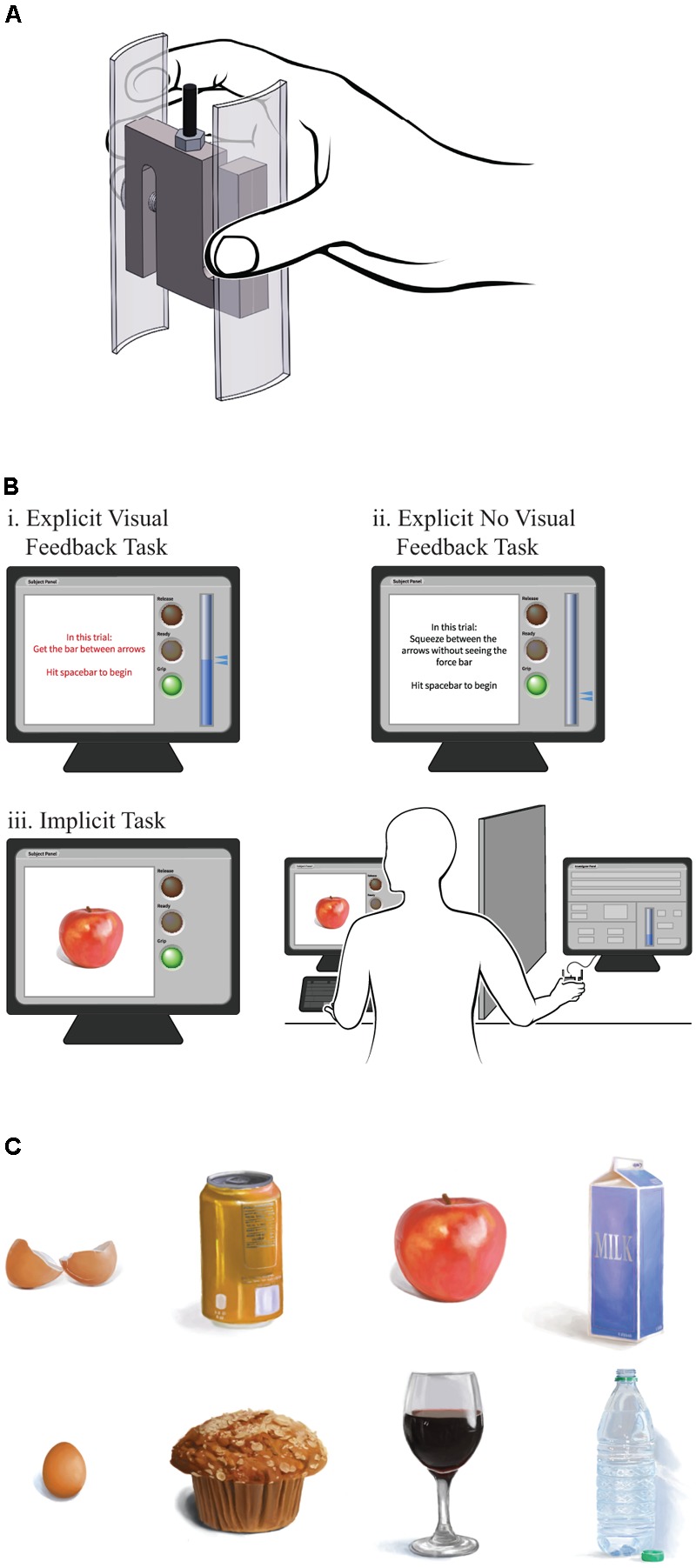
**(A)** Grasp manipulandum used for all tests, **(B)** experimental setup and participant screens for the three tasks, and **(C)** sample images used for the implicit task.

There appear to be three main studies on isometric force production and Fitts’ law in the extant literature that are relevant to the current approach. First, in a discrete aiming paradigm, Kantowitz and Elvers (1988) required participants to produce force with a joystick in order to move a video-display cursor into a target region. In that study, the target amplitudes and target widths were varied to provide three index-of-difficulty levels (3.5, 4.5, and 5.5 bits). In addition, the control-to-display gain (low or high) and the order of control (position or velocity) was varied. Regardless of the gain or order of control, the increases in “movement time” with the index of difficulty were reliable. Although there were only three index of difficulty levels it could be said that Fitts’ law held. Second, using a reciprocal aiming paradigm, Billon et al. (2000) instructed participants to move a video-display cursor between targets by producing (pushing and pulling) force on an isometric joystick using a precision grip. They (Billon et al., 2000) included 16 levels of the index of difficulty ranging between 2.74 and 6.44 bits and found that Fitts’ law provided a good fit to their data. The *r*^2^ value for the regression equation describing changes in the group-mean “movement time” as a function of the index of difficulty was 0.863 (Billon et al., 2000, see Figure 1, p. 50). That, along with their detailed analyses of the dynamics of cursor trajectories, lead the authors to conclude that Fitts’ law held for isometric as well as isotonic tasks and that “the chronometric and kinematic similarities between isotonic and isometric variants of Fitts’ aiming paradigm result from the presence of organizing constraints that operate at the level of the task.” (p. 51). Third, Kim et al. (2010) examined the production of isometric grip force production (using a power grip) in a reciprocal aiming paradigm. They were interested in using Fitts’ law to assess hand function in individuals with chronic stroke and how hand function might change as a function of practice. Performance was examined over five index of difficulty levels ranging between 2.5 and 5.0 bits, and participants performed the task over 12 sessions. When the Fitts’ law relation was examined on Days 1 and 11, the slope of the regression was reduced from 0.53 to 0.39—reflecting an improvement in performance—but, more importantly, the *r*^2^ value of the Fitts’ law relation was 0.98 in both cases. That is, Fitts’ law held both at the start and end of the study.

Like the study by Kim et al. (2010), the current study examined the applicability of Fitts’ law to the control of isometric grip force production in a targeted aiming task, when participants used a power grip to produce force. However, in the work presented here, force was produced using a discrete-trials procedure rather than a reciprocal aiming paradigm and we examined performance under different feedback conditions where the target amplitudes and target widths were both *explicitly* and *implicitly* defined. Under two of the three conditions, the target amplitudes [25, 50% maximum voluntary contraction (MVC)] and target widths (5, 10, 20, and 40% MVC) were explicitly defined on a computer monitor and the prescribed index of difficulty ranged between 0.70 and 3.46 bits. In one of those conditions, participants had continuous, online visual feedback of their force output in relation to the force target and in the other condition online visual feedback of force output was disabled while the force target was always visible. In the abovementioned studies on Fitts’ law and isometric force production, performance was only examined under conditions of continuous visual feedback (Kantowitz and Elvers, 1988; Billon et al., 2000; Kim et al., 2010). Furthermore, the majority of studies on isotonic control and Fitts’ law have only examined performance under continuous visual feedback conditions. Studies that have examined movements that were sufficiently rapid to expect minimal effects of vision (due to the relatively long latency for the processing of closed-loop visuomotor feedback) have found that spatial variability scales directly with movement amplitude and the imposed movement durations (e.g., Schmidt et al., 1979); since those movements are ultimately produced via force production, we would have expected to see similar results in our own conditions without visual feedback.

In this study, a comparison of the no visual feedback and visual feedback conditions provided us with the opportunity to examine the contribution of feedforward and feedback processes, respectively, to the control of isometric grip force. In a third condition, in an effort to move toward a more naturalistic and functional task, no visual feedback of force output was provided and participants were shown images of graspable everyday objects (e.g., an apple, an egg, a full wineglass, etc.) and were asked to produce force as if they were actually grasping those objects. These object images were an attempt to provide targets which more closely reproduced a naturalistic grasping task. Specifying target force explicitly, either numerically or graphically, may be less intuitive and may not fully represent the range of tasks typically encountered during daily living. In this third, implicit condition, the force production requirements were implied by the displayed image. In that case, force output could only be based on each participant’s internal representation of the force output needed to act on the object, and force output would be regulated through feedforward control processes and tactile feedback. In all three conditions of the study, we tested for compliance with Fitts’ law by calculating the *effective* index of difficulty, which was based on the effective amplitude and effective width (Welford, 1968; Crossman, unpublished). We use the effective adjustments for accuracy rather than prescribed values because the spread of end-point values may not perfectly align with the prescribed targets (Soukoreff and MacKenzie, 2004; Slifkin and Eder, 2017); participants may choose to take more time than necessary to hit the prescribed target, in order to aim for smaller regions at the nearer end of the prescribed targets (Soukoreff and MacKenzie, 2004; Slifkin and Eder, 2017); and specifically in the case of our implicit task, absolute prescribed values are not available nor particularly relevant. Thus, these effective values describe the targets, and thus the motor behavior, which the participants actually used. We compared the three conditions to examine changes in Fitts’ law as task information was modified.

Our goal with this study was to develop a procedure to quantify grasping performance in a realistic, functional task, represented by the implicit task (without visual force feedback and with naturalistic target objects). Such a procedure could have value for quantitative assessments of the performance of any type of grasper (biological, mechanical, or both) and could allow for comparisons between healthy normative grasping, grasping in various sensory-motor pathologies, and grasping with both conventional insensate prostheses and advanced sensorized prostheses. Such comparisons could facilitate improvements in decision-making about which approaches or interventions would have the greatest impact on functional performance. For example, the extent and nature of any deviations in the control of hand prostheses from control of the able-bodied hand can provide valuable information for improvements in prosthesis control and design.

We chose to focus primarily on the more naturalistic implicit task as an end goal for two main reasons. First, the scaling of motor output to the motor output requirements appears to occur with minimal visual attention, (Goodale et al., 1986; Frith et al., 2000) particularly when interacting with familiar objects (Frith et al., 2000). We suggest that the implicit task would be more similar to activities of daily living, where target forces are not explicitly specified; rather, target forces are implicitly understood via easily handled, familiar objects. Second, the implicit task would be expected to accommodate a range of motor performance abilities and operate without a ceiling effect because force output levels are self-selected and there are no explicitly imposed target widths.

## Materials and Methods

### Participants

Seventeen healthy adults (11 female, 6 male, 15 right-handed, 2 left-handed, average age 28 years, total age range 19–37 years) were studied under a protocol approved by the Institutional Review Board of the Cleveland Clinic and Department of Navy Human Research Protection Program. All participants were able-bodied, with no deficits in mobility or sensation in either of their upper limbs. Participants were naïve to the specific purpose of the experiment, but were told that they would need to repeatedly grasp a manipulandum to measure the speed and accuracy of their force production. Nine participants completed the explicit task first, with the other eight completing the implicit task first. The order had no significant effect (unpaired *t*-tests) on the primary measure of performance (throughput) in any of the conditions.

### Testing Apparatus

**Figure 1A** provides an illustration of the grip manipulandum, which we built from a single “s-type” 980 N capacity strain gauge force transducer and USB controller (Phidgets Inc., Alberta, Canada). Two 10.2 by 3.0 by 0.3 cm acrylic hand grips with a 3.5 cm radius of curvature were placed on the sides to constrain grip force along the primary axis of the load cell. This style of strain gauge is a rigid block that does not deform perceptibly during grip force production. We wrote software in LabVIEW (National Instruments, Austin, TX, United States, 2016) to run the experimental contingencies, which ran on a standard desktop computer during testing, and we used MATLAB (Mathworks, Natick, MA, United States, R2011a) to write the program for data analysis.

### Testing Procedure

**Figure 1B** provides an overview of the experimental setup. During the approximately 90-min testing period, participants sat at a desk with a keyboard and monitor, with the manipulandum placed on the desk within easy reach on their dominant side. We blocked the participants’ line of sight to the manipulandum with a barrier. We instructed the participants to place the fingers of their dominant hand around, but not touching, the manipulandum, and to rest the fingers of their non-dominant hand on the keyboard spacebar. Before the test, we asked participants to strongly grip the manipulandum for several seconds. We defined the average peak force of three such trials as their MVC. During the experiment itself the force targets and force output were normalized and expressed as percentages of the MVC. That ensured that all force requirements would be within each participant’s force production range and performance under a given force requirement would result in comparable levels of fatigue, if any, across participants. During the tasks we showed them a target (described below) on the computer monitor and when they were ready they pressed the spacebar to begin each trial. Participants were presented with a “traffic light” style ready-set-go countdown display on the monitor: a red light for 1 s, a yellow light for 1 s, and finally a green light accompanied by the display brightening and emitting an audible tone. We instructed participants to grasp the manipulandum using all five distal phalanges. Participants then attempted to produce grip forces as appropriate for the active target, and they pressed the spacebar again when their attempt at producing the appropriate force was complete. We instructed participants to be as quick and accurate as possible in reaching the target. They were given a 1-min break every 32 trials to mitigate fatigue.

It is important to note that, in contrast to the studies on isometric force production discussed in the Introduction, our explicit task used force values scaled to individuals’ MVC rather than absolute, fixed force values. Since the index of difficulty for Fitts’ law uses a dimensionless ratio of force values (A/W), the absolute force scale (e.g., Newtons, pounds, or MVC) is canceled. Differences in MVC between participants would therefore also cancel. A core assumption of Fitts’ law is that this ratio, rather than the specific scaling of A and W, is the relevant quantity (Fitts’, 1954). Thus, at least in theory, index of difficulty values are comparable between the experiments. In practice, there may be little difference between using fixed force values and scaling to MVC for a normative population of healthy adults producing typical forces because the range of MVC values would be less than an order of magnitude (Massy-Westropp et al., 2011). However, the difference could become significant in light of our longer-term goals of considering prostheses and disease states, which is why we chose to use values in the context of a dynamic range over fixed values. An additional consideration of this decision is that, while we used breaks to mitigate fatigue, any effects would be consistent across participants, if present.

In the two explicit tasks, the computer monitor displayed a vertical meter showing the grip force produced on the manipulandum, calibrated so that the top of the bar was each participant’s MVC value. In addition, during each trial the monitor displayed two arrows alongside the force meter which *explicitly* defined the range of target forces for that trial. The arrows indicated target widths of 5, 10, 20, or 40% of the MVC, centered on force amplitudes of either 25 or 50% of the MVC, resulting in eight possible target displays.

The on-screen instructions prior to each trial indicated whether or not that trial would include visual feedback. In the trials with visual feedback, the participant’s grip force was displayed to them in real-time (**Figure 1Bi**). In the no visual feedback trials, target arrows were displayed but the force-feedback bar remained empty regardless of the force produced on the manipulandum (**Figure 1Bii**). That is, no feedback of force output was provided. Targets were presented in blocks of eight, with each of the eight targets appearing once per block in random order. A minimum 1-min rest was required every four blocks. The blocks alternated between the visual feedback and no visual feedback conditions; a visual feedback condition block always occurred before a no visual feedback condition block. There were 20 visual feedback blocks interleaved with 20 no visual feedback blocks. In sum, we presented each unique target display within each of the two explicit condition 20 times, for a total of 320 trials within a duration of approximately 60 min.

The implicit task followed a similar procedure, except we removed the force-feedback bar and target arrows completely and replaced them with an image of an object (see **Figure 1Biii**). As depicted in **Figure 1C**, eight common, everyday objects were presented: an apple, a loaf of bread, an egg, an empty eggshell, a closed cardboard milk carton, a full plastic bottle without its cap, an unopened aluminum soda can, and a full stemmed wineglass. The size of object images on the computer monitor were scaled to provide a rough compromise between the size of the actual objects and the manipulandum. Participants were instructed to imagine that the manipulandum was the displayed object, and that they should grasp the manipulandum with enough force so they felt they could hold the object without dropping, crushing, or damaging it. For any object that the participant could conceive of grasping multiple ways (e.g., grasping the wineglass by the stem vs. the bowl, or imagining the milk carton as full vs. empty) we instructed them to consistently choose one way for every presentation of that object. Participants were instructed not to lift the manipulandum during task performance. In the implicit task, as in all task conditions reported in this study, the manipulandum was out of the participant’s view, the participant saw the target object on the monitor and initiated the traffic light countdown when they were ready, and they attempted to achieve target grip force quickly and accurately. Thus, for the implicit task, target forces were not explicitly specified, rather the displayed objects represented a range of different appropriate force levels. In other words, the implicit task required each participant to use their internal representation of each object and the appropriate force to grasp it, in the absence of visual feedback. We presented the objects in blocks of eight, with each of the eight objects appearing once per block in random order. Participants rested for at least 1 min every 32 trials as before, and in this case data was collected on 20 presentations of each object (160 trials), which took approximately 30 min.

### Data Analysis and Variable Definitions

We analyzed all three test conditions for each participant separately. Regardless of target type or presence of visual feedback, we parsed each trial in the same way. We defined the force amplitude of each trial as the change in force from the beginning of the trial (i.e., the end of the countdown) to the peak force. We defined the duration of the trial as the period from the onset of force production (defined as the time that force first exceeds 0.5% MVC after the countdown) until the peak force was reached. During data analysis, individual trials were discarded if the force at the beginning of the trial exceeded 0.5% MVC (e.g., the participant grasped the manipulandum early), if the force during the trial never exceeded 1.0% MVC (e.g., the participant ended the trial without grasping the manipulandum), or if the researcher noted any invalidating behavior during the trial (e.g., if the manipulandum slipped from the participant’s grasp, or the participant stopped to ask a question during a trial). On average, 2.9% (3.4% SD) of the trials were discarded, with no significant differences (paired *t*-tests) among the test conditions. For each of the three task conditions, trials were collated by distinct target display, and then any trial whose duration or amplitude fell outside three standard deviations was discarded as an outlier. The outliers comprised 1.0% of the otherwise valid trials; paired *t*-tests revealed no significant differences between test conditions.

For each target display (i.e., the eight force-meter target displays and eight object images), we defined the *effective* amplitude and *effective* width values of that target based on the distribution of peak forces generated during all presentations of that target display. The effective amplitude (Ae) was simply the mean of the distribution of peak forces, and the effective width (We) was calculated from the standard deviation of the distribution (Welford, 1968; Crossman, unpublished), as

(1) $$We=\sqrt{2\pi e\sigma^{2}}=4.133\sigma$$

This effective width therefore describes the size of the target region each participant actually used 96% of the time. The effective values for both amplitude and width reflected the range of forces that participants effectively used and were used in the calculation of an effective index of difficulty (IDe).

We calculated IDe using the Shannon formulation (MacKenzie, 1989)

(2) $$IDe=log_{2}\left(\frac{Ae}{We}+1\right)$$

The same formula can be used to obtain the prescribed, as opposed to effective, index of difficulty (ID) using amplitude (A) and width (W) of a target. The prescribed indices of difficulty presented in the explicit task with and without visual feedback are listed in **Table 1**. We used the Shannon formulation both because of its well-documented (MacKenzie, 1989, 1992; MacKenzie et al., 1991) advantages over the Fitts’ (1954) and Welford’s (1960) formulations of the index of difficulty. In particular, Equation 2 guarantees a positive value for IDe (an important property for calculating throughput, see below) even in cases where the effective width is much larger than the effective amplitude (i.e., where the effective width encompasses the starting position). As part of the current effort to develop a method for assessing performance differences between individuals (e.g., able-bodied performance vs. those with prosthetic limbs), we considered the maximum IDe (the highest target IDe value each participant produced) to be of particular interest, as it represented the upper limit of the relative precision domain within which each participant operated. This is because an average IDe would include object interactions where fast-but-imprecise operation represents a choice of the operator, rather than a limitation of the system. The mean time to peak force (TPF) for each target display is the mean duration of its valid trials. For each set of target displays, we calculated throughput (TP) (Soukoreff and MacKenzie, 2004) as

**Table 1:** Prescribed amplitude, width, and ID for the explicit task.

A (% MVC)	W (% MVC)	ID (bit)
25	5	2.58
25	10	1.81
25	20	1.17
25	40	0.70
50	5	3.46
50	10	2.58
50	20	1.81
50	40	1.17

(3) $$TP=\frac{1}{n}\sum_{\text{i=1}}^{\text{n}}\frac{IDe_{\text{i}}}{TPF_{\text{i}}}$$

For comparison, the data were also fit by a conventional Fitts’ law least-squares linear regression,

(4) TPF=a+b×IDe

The inverse slope of the linear regression (*b*^-1^) and throughput are similar quantities that describe the same underlying property: the index of performance (or information processing capacity) of the system, measured in bits per second. Throughput is preferred here because it combines the effects of intercept and slope of the regression into a complete measure encompassing both the speed and accuracy of performance (Soukoreff and MacKenzie, 2004) and more importantly because it is a more robust calculation. This robustness is important when, for example, the domain of the data is small, as is expected to be the case for individual participant data. In this case, noisy measurements over the small domain are likely to cause a poor linear regression, yielding unreliable regression coefficients. Since throughput relies on the assumption that Fitts’ behavior has already been demonstrated, we needed to confirm that Fitts’ law applied to the overall group behavior, so we averaged the group data to reduce variability and allow linear regression coefficients to be calculated. However, due to the robustness of throughput, that is the method that would necessarily be used to evaluate performance of a single participant since regression coefficients are not guaranteed (or even likely in our case) to be robust for an individual participant. The ability to perform these calculations at the individual level (as opposed to just the group-mean level) is an important goal of our study, which is to make this a useful diagnostic tool.

### Statistical Methods

We assessed potential differences for throughput and maximum IDe across the three target conditions (explicit with vision, explicit without vision, and implicit) using one-way repeated-measures ANOVAs, with *post hoc* tests determining significance between the different target conditions. We examined the consistency of throughput performance between the tests with Pearson’s correlations.

We investigated the influence of various factors on effective amplitude and effective width. For the explicit task, we used full-factorial three-way repeated-measures ANOVAs to determine the influence of feedback (vision vs. no vision), amplitude (25 and 50% MVC), and width (5, 10, 20, and 40% MVC). We investigated significant interaction effects involving feedback with *post hoc t*-tests. For the implicit task, we used one-way repeated measures ANOVAs to determine the influence of the targets (eight images). All *post hoc t*-tests were Bonferroni-corrected (α = 0.05). All *t*-tests were two-tailed, paired tests, and all means are presented as ±1 standard deviation, except where otherwise stated. We tested for sphericity using Mauchly’s test, and if the sphericity assumption was violated we used Greenhouse–Geisser adjusted *F*-tests, an adjustment which reduces the risk of false positive results. We reported these adjusted degrees of freedom rounded to the nearest whole number.

## Results

### Individual Level Performance

We prescribed grip force targets to participants, both explicitly with arrows on a force meter (with and without visual force-feedback from the meter, **Figures 1Bi,ii**), and implicitly by presenting images of everyday objects (**Figure 1Biii**), which they imagined grasping. **Table 2** summarizes the average MVC, throughput, and maximum IDe of all participants in the three conditions. Throughput and maximum IDe are highest for the explicit task with visual feedback, substantially lower for the explicit task without visual feedback, and slightly lower still for the implicit task [throughput *F*(2,27) = 93.13, *p* < 0.0001, η^2^ = 0.60; maximum IDe *F*(1,21) = 138.04, *p* < 0.0001, η^2^ = 0.84; pairwise tests for both measures, explicit task with vision vs. other conditions, *p*-values < 0.0001, explicit task without vision vs. implicit task, *p*-values ≤ 0.018].

**Table 2:** Participants’ average MVC, throughput, and maximum IDe.

Target condition	MVC (N)	TP (bit/s)	Max IDe (bit)
Explicit task with vision	52.0 ± 21.6	3.43 ± 0.80	3.17 ± 0.63
Explicit task without vision	52.0 ± 21.6	1.79 ± 0.85	1.43 ± 0.22
Implicit task	52.0 ± 21.6	1.39 ± 0.55	1.16 ± 0.22

For all three test conditions, the average maximum IDe each participant generated (**Table 2**) was less than the maximum prescribed ID in the explicit conditions (3.46 bits, **Table 1**). The average minimum IDe values generated in the explicit task with and without visual feedback were 1.25 and 0.96 bits, respectively (not tabulated), both of which were greater than the minimum prescribed ID (0.70 bits, **Table 1**). The average minimum IDe from the implicit condition of 0.68 bits (not tabulated) was comparable. Taken together, while our total prescribed ID range was narrower than what is commonly used in spatial pointing applications of Fitts’ law, it seems that it was sufficient to encompass common grasping force behavior.

Given the narrow range of IDe values produced, and the limitations of the linear regression method, throughput is the preferred metric, as discussed in Section “Data Analysis and Variable Definitions.” However, for comparison, we also calculated the linear regression for each participant’s data (Eqn. 4). Only the explicit task with vision generated consistently high *r*^2^ values (0.82 ± 0.08). The intercepts (*a* = 0.019 ± 0.188 s) were small and within the range expected from a valid Fitts’ law model (typically -0.02 to 0.04 s, Soukoreff and MacKenzie, 2004). The mean slope (*b* = 0.304 ± 0.125 s/bit) of the individual-participant linear regressions agreed well with their corresponding average throughput values (0.304^-1^ ≈ 3.43 bits/s), which is expected given the consistently high individual-participant *r*^2^ values for the regression in this condition. Without visual feedback, the *r*^2^ of the linear regressions for the explicit task without vision (0.18 ± 0.23) and the implicit task (0.28 ± 0.24) were low, meaning each individual participant’s performance on these two tasks was not well explained by the regression. Values for individual regression coefficients (slope and intercept) were calculable, but with the poor fit of the linear model to these data, we did not consider the regression coefficients to be reliable.

### Group Level Performance of Explicit Task

**FIGURE 2:**
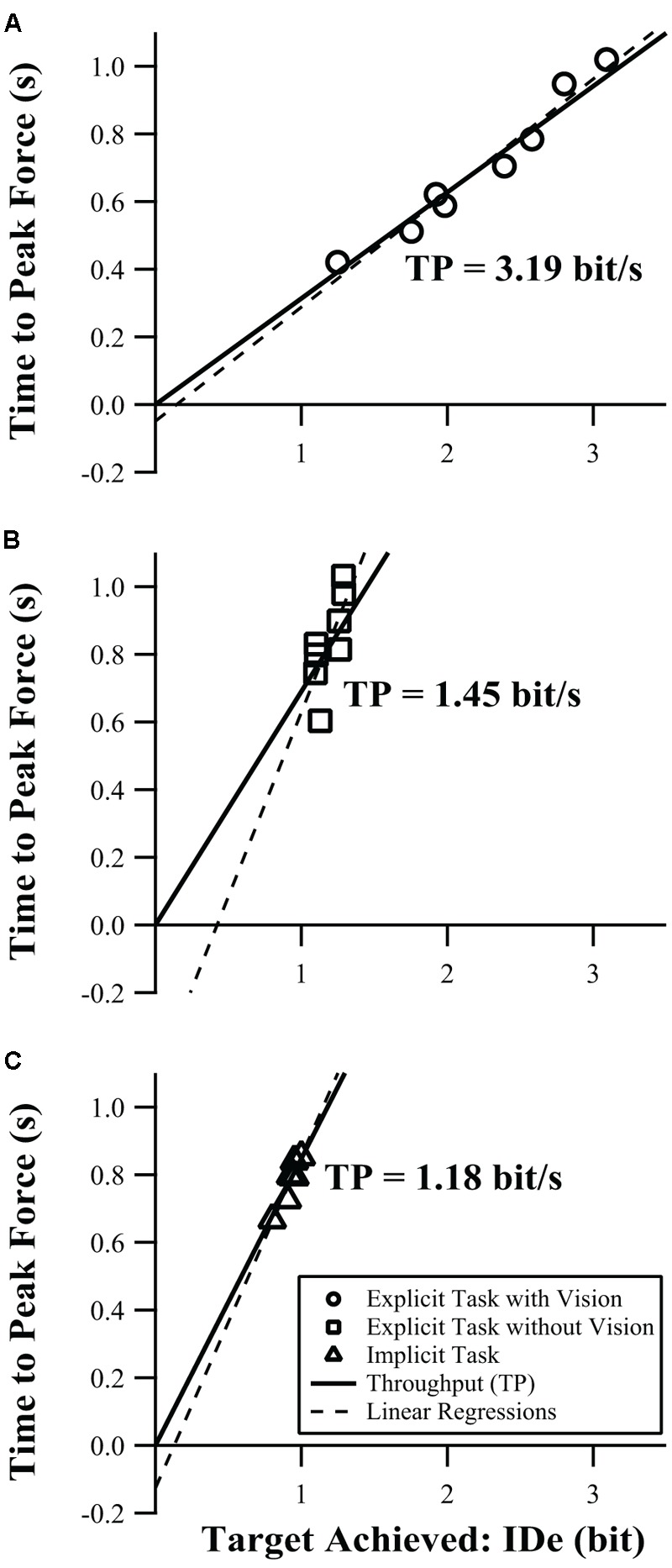
Time to peak force and IDe values shown are averaged across all participants. Solid lines show throughput in bit/s, where TPF = TP ^∗^ IDe; dashed lines represent linear regressions as follows: **(A)** Explicit task with Visual feedback: TPF = 0.337 ^∗^ IDe + -0.049, *r*^2^ = 0.96; **(B)** Explicit task without visual feedback: TPF = 1.089 ^∗^ IDe + -0.460, *r*^2^ = 0.54; **(C)** Implicit task: TPF = 0.982 ^∗^ IDe + -0.127, *r*^2^ = 0.89.

We also examined the overall behavior of the population, which was accomplished by averaging the IDe and time to peak force of each target across all participants. This produced an average group level performance for each task, and we performed linear regression and calculated throughput for these averaged data for the explicit task with visual feedback, the explicit task without visual feedback, and the implicit task. The group level performance for the three tasks is summarized in **Figure 2**, which represents the approach of averaging across individuals and calculating the regressions of those averages. This group level approach is important to consider because it reduces variability and allows a clearer view of the underlying trends, and can confirm that otherwise noisy individual-participant behavior is following Fitts’ law. In contrast, **Table 2** represents the individual level approach of calculating the regression of each individual, and averaging those regression parameters, which, while subject to more measurement noise, is more representative of how this test would be used in practice. At the individual level, only throughput was a robust calculation, but at the group level, both linear regression and throughput are usable and roughly equivalent. However, due to the lack of robustness at the individual level where any test of function would necessarily be applied, we used throughput as the primary index of performance throughout.

The explicit task with visual feedback is the most analogous to a conventional pointing task because, in this condition, the manipulandum functions as a one-degree-of-freedom pointer, and the force feedback is displayed visually as a cursor. Changing only the nature of control from isotonic pointing to isometric force production makes for a logical stepping off point toward the more naturalistic implicit task, before manipulating feedback and target type, because this preserves the other aspects of a conventional Fitts’ paradigm while changing only the nature of the “pointing” task from position in space to grip force. The group level performance for the explicit task with visual feedback is shown in **Figure 2A**. As throughput (Eqn. 3) describes the first-order (linear) relationship between TPF and ID, it can be visualized as a straight line passing through the origin, with slope equal to TP^-1^, shown as a solid line in **Figure 2A**. The throughput and regression (dashed line) are similar, the *r*^2^ of the regression is high (*r*^2^ = 0.96), and the intercept is small, confirming that Fitts’ law provides a good model of performance in the isometric force production task with visual feedback.

In practical grasping tasks, informative visual feedback is not required to successfully grasp an object (Frith et al., 2000). The explicit task without visual feedback removes the contribution of vision to the control of motor output in the task, and the average performance is shown in **Figure 2B**. The higher slope of both the throughput and linear regression show that the quality of the speed-accuracy tradeoff is diminished without visual feedback. The *r*^2^ value of the linear regression for the group-mean is lower without vision (*r*^2^ = 0.54), and its intercept is outside the -0.2 to 0.4 range which is generally considered acceptable (Soukoreff and MacKenzie, 2004). A possible explanation for the reduction in the *r*^2^ value, at least in part, is that the IDe values are all similar to each other, i.e., the narrow domain issue discussed in Section “Data Analysis and Variable Definitions.” Briefly, linear regression is valid only over the domain of the data, and as the domain becomes increasingly narrow the fit of the regression becomes more sensitive to small variation (i.e., less robust), even if the underlying relationship is linear.

**FIGURE 3:**
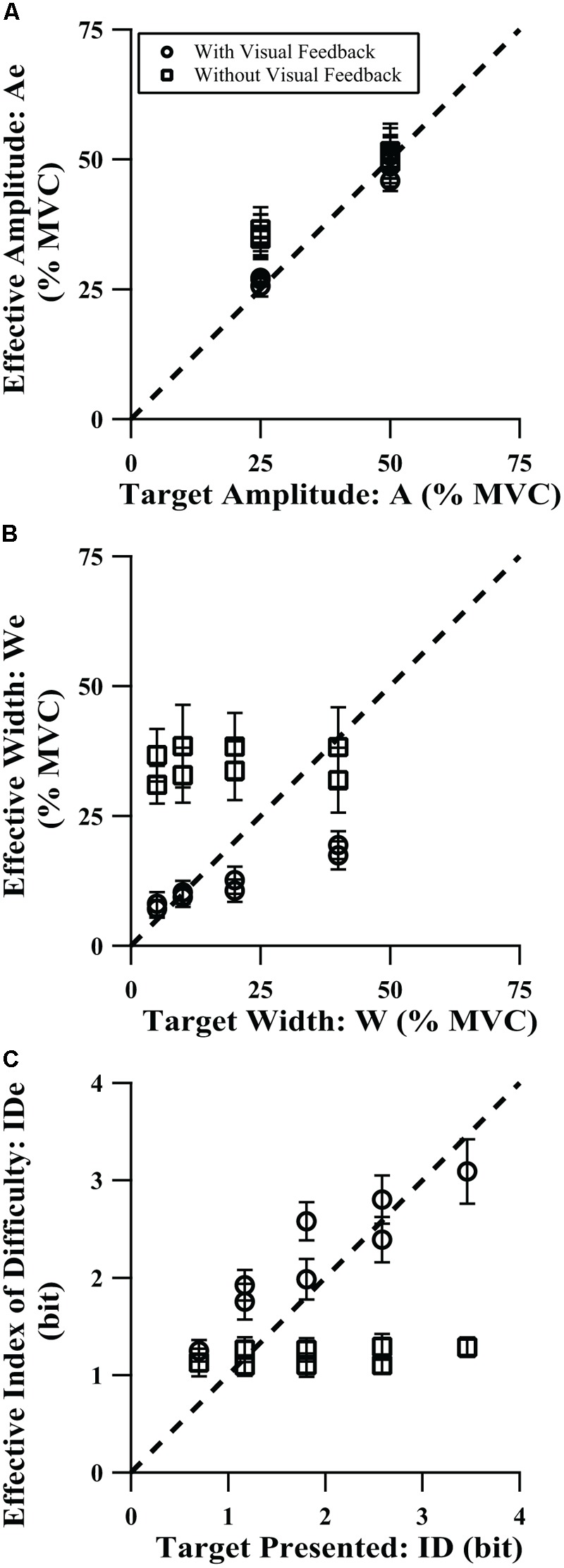
Comparison of effective vs. presented target values for **(A)** amplitude, **(B)** width, and **(C)** index of difficulty, for the explicit target tasks. Error bars show 95% confidence interval.

We wanted to determine the influence of target amplitude, width, and presence of visual feedback on the effective target display values (Ae, We, and IDe). **Table 3** summarizes the explicit targets’ prescribed target amplitude and target width condition as a percentage of MVC and their ID, the participants’ average effective values in the visual feedback and no visual feedback conditions, and the time to peak force for each. The three-way (feedback by target amplitude by target width) ANOVA was used to assess changes in the effective amplitude as a function of those factors. As shown in **Figure 3A**, we found that average force amplitude scaled relatively well to target amplitude without vision, although the loss of vision caused them to squeeze harder at the low amplitude (*p* = 0.0005 across all target widths for 25% MVC). We found a significant main effect of feedback on effective amplitude [*F*(1,16) = 6.20, *p* = 0.024, η^2^ = 0.045]. When the target amplitude increased, the group-mean effective amplitude increased [*F*(1,16) = 377.05, *p* < 0.0001, η^2^ = 0.61], with a small but significant target amplitude by target width interaction [*F*(2,35) = 11.87, *p* < 0.0001, η^2^ = 0.0033, **Table 3**] and a significant feedback by target amplitude interaction [*F*(1,16) = 18.49, *p* = 0.0006, η^2^ = 0.023]. Across test conditions, there was a small but significant main effect of target width on effective amplitude [*F*(1,24) = 8.92, *p* = 0.0027, η^2^ = 0.0053], with a significant feedback by target width interaction [*F*(2,29) = 5.56, *p* = 0.011, η^2^ = 0.0018], although the values were largely similar within amplitude and feedback combinations. Furthermore, the target amplitude influenced the effective amplitude regardless of visual feedback condition (pairwise comparisons *p*-values < 0.0001), while width only influenced effective amplitude in the presence of visual feedback (pairwise comparisons *p*-values ≤ 0.023). Collectively, this manifests in **Figure 3A** as clusters of effective width requirements at different target amplitudes that are largely indistinguishable from each other; higher effective amplitudes without visual feedback, particularly for the lower target amplitudes; and substantially higher effective amplitudes for higher target amplitudes, with particularly good agreement with the target amplitude with visual feedback and at the 50% MVC target amplitude.

**Table 3:** Participants’ performance during explicit task.

% MVC						Bit			Millisecond	
A	Ae vision	Ae no vision	W	We vision	We no vision	ID	IDe vision	IDe no vision	TPF vision	TPF no vision
25	26.9 ± 1.1	34.7 ± 7.4	5	7.1 ± 3.6	31.0 ± 7.6	2.58	2.39 ± 0.49	1.10 ± 0.18	705 ± 254	830 ± 378
25	27.2 ± 1.5	35.5 ± 8.2	10	10.3 ± 4.7	32.8 ± 11.2	1.81	1.98 ± 0.44	1.10 ± 0.25	588 ± 199	798 ± 408
25	27.0 ± 2.1	36.6 ± 8.9	20	12.6 ± 5.5	33.7 ± 11.9	1.17	1.75 ± 0.38	1.10 ± 0.22	512 ± 167	745 ± 434
25	25.6 ± 4.2	35.1 ± 9.1	40	19.4 ± 5.6	31.9 ± 13.2	0.70	1.25 ± 0.23	1.13 ± 0.30	420 ± 133	603 ± 311
50	51.4 ± 1.1	51.2 ± 10.2	5	8.1 ± 4.7	36.7 ± 10.6	3.46	3.09 ± 0.70	1.29 ± 0.21	1020 ± 395	1030 ± 495
50	50.7 ± 1.4	51.6 ± 11.1	10	9.4 ± 3.9	38.4 ± 16.7	2.58	2.80 ± 0.52	1.29 ± 0.28	947 ± 323	979 ± 586
50	48.7 ± 1.6	50.1 ± 9.8	20	10.6 ± 4.6	38.4 ± 13.6	1.81	2.58 ± 0.41	1.26 ± 0.26	784 ± 240	898 ± 444
50	45.9 ± 4.1	49.5 ± 10.0	40	17.4 ± 5.7	38.3 ± 16.1	1.17	1.92 ± 0.33	1.26 ± 0.27	621 ± 187	814 ± 423

**Figure 3B** shows that with vision, participants’ effective width increased as the target width increased, although not as quickly as the target width increased, and the effective width was higher for the no visual feedback condition. That gave rise to a significant main effect of target width [*F*(2,33) = 10.04, *p* = 0.0003, η^2^ = 0.020] and a significant main effect of feedback condition [*F*(1,16) = 119.58, *p* < 0.0001, η^2^ = 0.57]. In addition, there was a significant feedback condition by target amplitude interaction [*F*(1,16) = 9.47, *p* = 0.0072, η^2^ = 0.011, **Table 3**] and a significant feedback condition by target width interaction [*F*(2,37) = 9.15, *p* = 0.0001, η^2^ = 0.016]. As seen in **Figure 3B**, effective width increases when target width increases, but only when there is visual feedback, which led to the feedback condition by target width interaction. In the no vision condition, participants adopted a common, large effective width under all target widths. With vision, effective width increased with target width for all except the 10–20% MVC comparison (non-significant increase at *p* = 0.054, *p*-values ≤ 0.0003 for all others in *post hoc t*-tests). At the wider target widths, participants tended to produce narrower effective widths than prescribed, i.e., their force variability was less than what was permitted by those targets (see Slifkin and Eder, 2017, Figure 3, for a similar pattern of results in a cyclical aiming task). Without vision, despite the significant main influence of target width on effective width, it was not systematic without vision and pairwise comparisons were non-significant. Participants’ force variability was consistently higher (pairwise *p* = 0.028) at the higher force requirement (50% vs. 25% MVC) without vision.

**Figure 3C** combines Ae and We into IDe (Eqn. 2). We found that with vision, participants achieved higher effective difficulty values than was prescribed for easy targets, but IDe matched the prescribed ID values more closely for more difficult targets. An additional ANOVA for IDe was not performed for two reasons. First, within each ID level in each feedback condition, there was either one or two versions of that ID level, and that imbalance would make it difficult to easily fit the data into the three-way ANOVA framework used for effective amplitude and effective width. Second, even if an ANOVA were possible, because IDe was derived only from Ae and We, and any significant changes in IDe would be entirely due to Ae and We, an additional analysis of their combined effect creates risk of type I error.

### Group Level Performance on Implicit Task With Naturalistic Targets

We were concerned that explicit targets (i.e., arrows on a force meter) would not be analogous to grasping rigid objects in daily living. Therefore, we designed the implicit task to be more natural with the force requirements implied by familiar, everyday objects for which the participants would be expected to have well-established internal representations. An important feature of the implicit targets was that they had no prescribed requirements for force amplitude, target width, and ID: the amplitude and width requirements for handling any given object were ultimately defined by each participant. We used the effective amplitude, effective width, and IDe to characterize performance, just as was done for analyses of performance of the explicit tasks. Although absolute, prescribed values could be obtained for real-world objects, it only mattered how each participant interacted with each object.

Understanding the target itself appears to be an important determinant of the fit of Fitts’ law to the data. For the group-mean data, we found that even without vision the fit of Fitts’ law improved for the implicit target condition as compared with the explicit target condition without vision (**Figures 2C** vs. **2B**). The range of IDe was compressed, similar to the explicit task without visual feedback (IDe values span a range of 0.20 vs. 0.19, respectively), but compared to the explicit task without feedback the regression was more linear over that range (*r*^2^ = 0.89 vs. 0.54, respectively), the regression matched the throughput line more closely, and the regression intercept was improved (-0.127 being closer to zero than -0.460). Although the range of IDe values was narrow, it appeared to follow Fitts’ law.

**FIGURE 4:**
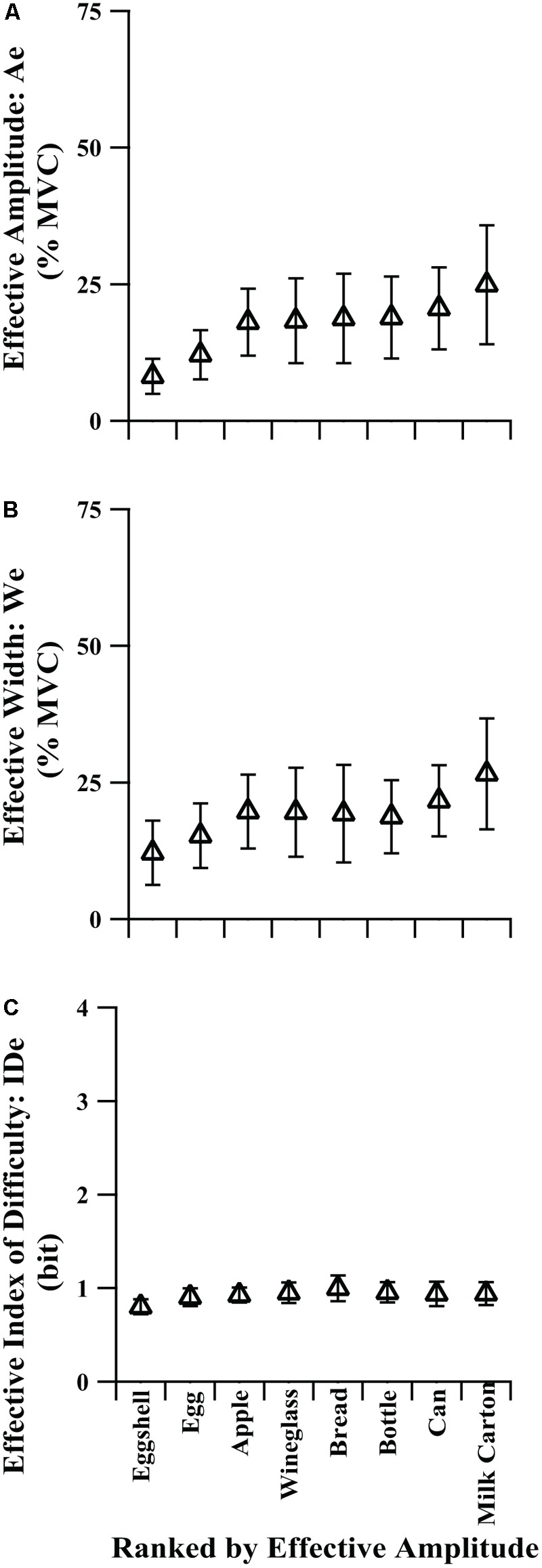
Comparison of effective target values for **(A)** amplitude, **(B)** width, and **(C)** index of difficulty vs. implicit target in ranked order by effective amplitude. Error bars show 95% confidence interval.

We needed to determine the influence of the implicit targets (images) on the effective targets, summarized in **Table 4** and shown in **Figure 4**. **Figure 4** can be considered analogous to **Figure 3**, except without absolute target values as an independent variable. Instead, all target objects in **Figure 4** were placed in rank order according to their effective amplitude values. We found that the average effective amplitudes of the implicit targets were generally lower than those for the explicit task; the effective amplitude for the milk carton approached 25% MVC (**Figures 3A, 4A**). Furthermore, the implicit task generated more precise grip forces than in the explicit task without vision with some average effective target widths falling within the range of the visual feedback condition. This was evidenced by the effective width values for the natural targets (**Figure 4B**) being lower than those in the no visual feedback condition (**Figure 3B**) and overlapping with the effective width values from the visual feedback condition (**Figure 3B**). Across the group of individual participants, effective amplitude ranged from 1.1 to 73.4% MVC and effective width ranged from 1.2 to 74.3% MVC. Despite the wide range of peak force amplitudes and the range of force variability across participants, the average IDe was relatively consistent across the target images, and the individual participants’ target IDe values spanned only 0.47–1.65 bits. In addition, one-way repeated-measures ANOVAs applied to the data in **Table 4** and **Figure 4** revealed that the image presented to the participants (target object) significantly influenced both the average effective amplitude [*F*(2,28) = 9.15, *p* = 0.0013, η^2^ = 0.095] and the average effective width [*F*(3,53) = 5.44, *p* = 0.0018, η^2^ = 0.063].

**Table 4:** Participants’ performance during implicit task.

Object	Ae (%MVC)	We (%MVC)	IDe (bit)	TPF (millisecond)
Eggshell	8.1 ± 6.8	12.1 ± 12.4	0.80 ± 0.17	666 ± 379
Egg	12.1 ± 9.4	15.3 ± 12.5	0.90 ± 0.20	726 ± 355
Apple	18.0 ± 12.9	19.7 ± 14.4	0.93 ± 0.17	797 ± 329
Wineglass	18.3 ± 16.3	19.5 ± 17.2	0.95 ± 0.23	840 ± 451
Bread	18.7 ± 17.2	19.3 ± 18.7	1.00 ± 0.29	852 ± 429
Bottle	18.9 ± 15.8	18.7 ± 14.0	0.96 ± 0.22	794 ± 352
Can	20.6 ± 15.7	21.6 ± 13.7	0.94 ± 0.27	794 ± 348
Milk Carton	24.9 ± 22.9	26.6 ± 21.3	0.94 ± 0.26	799 ± 333

## Discussion

We found that isometric grip forces continued to follow Fitts’ law both without visual feedback and also when conventional targets were replaced by implicit targets in the form of images of everyday objects. The ability to apply this same model to the implicit task allows for an already well-understood framework to describe the functional performance of a grasper in a naturalistic task that more closely resembles activities of daily living.

In an effort to understand the underlying behavior, we averaged all participants’ IDe and TPF values and modeled them as a single unit (**Figure 2**) and compared that to the results from the seventeen individual participants (**Table 2**). We found that for the explicit target visual feedback condition, both the throughput and regression coefficients of the average participant were similar to those of the individual participants (within 2 SEM), and the *r*^2^ for the linear regression was improved from 0.82 ± 0.08 for the individuals to 0.96 for the averaged targets. In the two tasks without visual feedback, the ranges of IDe values were compressed enough to compromise the interpretation of the linear regression. However, the reduced noise of the averaged response allowed for a more reliable fit. Modeling the group-mean IDe-TPF relation in the no visual feedback explicit target condition yielded an improved *r*^2^ value (0.54 vs. 0.18). Moreover, the implicit target model for the group-mean data, while still restricted to a narrow IDe range like that of the individual participants, was highly linear (*r*^2^ = 0.89 vs. 0.28). This suggests that information processes governing motor output still comply with Fitts’ law, despite the removal of visual feedback and change of target type.

**FIGURE 5:**
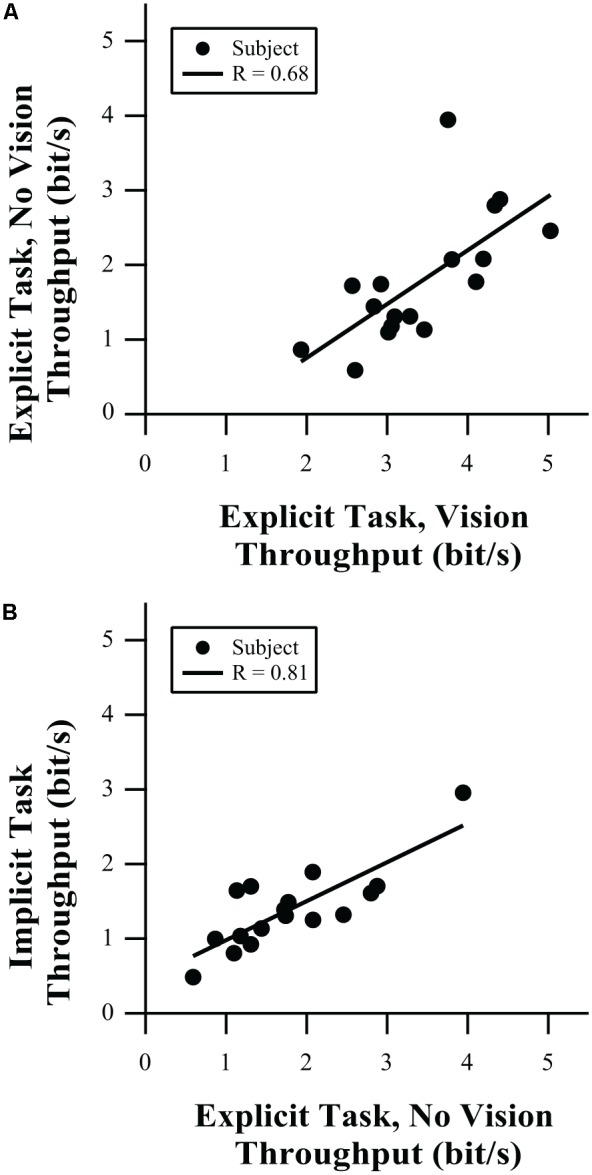
Correlation of throughput in **(A)** the explicit task, with vs. without visual feedback, and **(B)** implicit task vs. explicit task without vision.

Throughput does not have the same limitations as linear regression; as long as the implied relationship has a small intercept (confirmed by the group-mean data presented in **Figure 2**) and the data are not clustered at the origin (guaranteed by the choice of using the Shannon Formula for IDe). **Figure 5** shows correlations in throughput between tests. An individual data point in that figure represents the average throughput for an individual participant in one test condition plotted against the corresponding throughput value in another condition. In both figures, we see that individuals with low throughput in one task tend to have low throughput in the other task and those individuals with high throughput in one task have high throughput in the other task. As shown in **Figure 5**, throughput in the explicit task with and without vision is correlated (*R* = 0.68, *p* = 0.0027), so while there are substantial differences in performance with and without vision, participants’ relative information processing capacity on the two tests was preserved. Further, comparing the explicit task without vision to the implicit task (**Figure 5B**) showed significantly correlated behavior (*R* = 0.81, *p* < 0.0001), even though the implicit targets generated lower levels of grip force variability (**Figures 4B** vs. **3B**) and more linear behavior (**Figures 2C** vs. **2B**). It is hypothesized that it is the feedforward component of the control of force output that drives those correlations in information processing capacity. Support for that hypothesis may be apparent in the stronger correlation of throughput for the explicit task without vision and the implicit task. In that case, there is no visual feedback to be processed, which would suggest feedforward control as a main source of motor output regulation. However, correlation for the explicit task with visual feedback and the explicit task without visual feedback is reduced. That may reflect the additional influence of visual feedback on throughput when vision was available, which would not be available when feedback was removed.

Our implementation of the manipulandum imposed certain constraints on the participants’ grasp and the target objects in the implicit task. The manipulandum was incompressible, so isometric grip force (in the absence of visual feedback) was based on effort and modulation of that effort through tactile feedback. The objects were selected to be roughly the size of the manipulandum, and somewhat rigid, at least in the case of a “successful” (i.e., not crushing) handling. Because we did not want to continually remind participants of the manipulandum’s actual weight, we instructed them to grasp the manipulandum such that they *could* lift the object, but to not actually lift the manipulandum. We hoped that these considerations would minimize conflicting information from the manipulandum that might interfere with their imagining that the objects were being grasped. It is likely that adding an additional feedback modality could improve performance; a more sophisticated manipulandum (with variable weight or compressibility for different targets) could be used to examine these effects.

Of the two conditions without visual feedback (explicit task without vision and implicit task), the group-mean implicit task IDe-TPF relation was better described by linear regression. This poorer description of the explicit task without vision by Fitts’ law can also be seen as greater deviation of individual points from the throughput line in **Figure 2B**. A possible explanation for the decreased performance on the explicit task without visual feedback is that the targets were somewhat abstract, e.g., the average person likely does not have an identifiable percept for what 25 ± 5% of their MVC feels like, so while participants did well in scaling their average peak force values to the prescribed target amplitudes (Ae vs. A, **Figure 3A**), their trial-to-trial variability was quite high and constant across target width levels (high We values, **Figure 3B**). In the absence of vision, participants relied on memory and internal representation of how to meet the amplitude and target width requirements associated with each target display. In terms of motor-output variability theory, participants may execute a pre-selected motor pattern to the best of their ability, but without vision they have trouble scaling force variability (We) to the target width (W) separately from force output level specified by the motor command (Ae). In the current study, force variability, We, and the average force produced, Ae, are not completely proportional without visual feedback as one might expect based on rapid movements (Schmidt et al., 1979), but the influence of A on We in the condition without visual feedback does imply substantial proportionality, resulting in the relatively flat values of IDe generated in those conditions. This is reflected in **Figure 3**, where the primary difference in the explicit task with and without vision appears to be a reduced ability of participants to tailor the variability of their force output to the variability limits imposed by the target boundaries when vision was absent; vision seems to be one way to correct for such variability during execution of the grasp. The ability to generate grip force amplitudes matching the target amplitudes, while affected, remains largely intact. This suggests that the initial feedforward component of the action is relatively solid. In contrast, the implicit targets represented everyday objects for which participants would be expected to have an intrinsic understanding of their properties. As such, they were able to scale both grip force amplitudes and widths for different objects despite the lack of any visual force feedback (**Figure 4B**). The force variability data in **Figure 4B** still show some proportionality to the force amplitude data of **Figure 4A**, but allow for significantly different, if still relatively flat, IDe values shown in **Figure 4C**.

Amplitude and widths can be successfully scaled in the implicit task, but for the everyday objects in the task these quantities tended to scale together, resulting in a relatively narrow range of IDe values. With the demonstration that the limits of IDe could be directly and consistently reached, it provides evidence that this limit could be used as a descriptor for grasping ability. Combining throughput and maximum IDe shows promise for allowing comparisons of grip force control, motor system deficits, grip conformations, and grasping devices across individuals.

## Ethics Statement

This study was carried out in accordance with the recommendations of the Institutional Review Board of the Cleveland Clinic and Department of the Navy Human Research Protection Program, with written informed consent from all subjects in accordance with the Declaration of Helsinki. The protocol was approved by the Institutional Review Board of the Cleveland Clinic and Department of the Navy Human Research Protection Program.

## Author Contributions

ZT, AS, DB, and PM contributed to the design of the study. ZT and DB designed the software and collected and analyzed the data. All authors contributed to the manuscript and approved the final version.

## Conflict of Interest Statement

The authors declare that the research was conducted in the absence of any commercial or financial relationships that could be construed as a potential conflict of interest.
